# Identification of Maternal Serum Longitudinal Signatures Through Profiling of 96 Cytokines

**DOI:** 10.3390/life16050710

**Published:** 2026-04-22

**Authors:** Viktoriia Kolesnyk, Colleen Sinnott, Tobias Max Philipp Hartwich, Foram Gandhi, Jennifer Culhane, Lisbet Lundsberg, Sonya Abdel-Razeq, Miranda Mansolf, Samantha Novo, Olga Grechukhina, Yang Yang-Hartwich

**Affiliations:** Department of Obstetrics, Gynecology, and Reproductive Sciences, Yale University, New Haven, CT 06510, USA; viktoriia.kolesnyk@yale.edu (V.K.); colleen.sinnott@yale.edu (C.S.); tobias.hartwich@yale.edu (T.M.P.H.); foram.gandhi@yale.edu (F.G.); jennifer.culhane@yale.edu (J.C.); lisbet.lundsberg@yale.edu (L.L.); sonya.abdel-razeq@yale.edu (S.A.-R.); miranda.mansolf@yale.edu (M.M.); samantha.novo@yale.edu (S.N.)

**Keywords:** maternal serum, cytokines, multiplex cytokine panel, pregnancy, preeclampsia

## Abstract

Changes in the maternal serum cytokine landscape occur throughout pregnancy, representing immune adaptations and modifications to support a healthy pregnancy. A better understanding of normal cytokine patterns can help improve the management of pregnancy and potentially predict complications. Using maternal serum samples collected at four different timepoints from 29 subjects, we characterized the longitudinal serum cytokine patterns throughout normal pregnancy with a multiplex ELISA of 96 cytokines. Based on unsupervised principal component analyses of the 96-cytokine data, we developed an integrated panel that incorporated the data of 21 key cytokines. This integrated immune signature allowed us to distinguish serum samples collected at different stages of healthy pregnancy and evaluate their chemotactic properties in vitro. We also evaluated the potential of using integrated cytokine scores for identifying pathological condition like preeclampsia before clinical signs are presented. This study explored new approaches of developing serum biomarker panels for immune profiling and early detection of pathological conditions during pregnancy.

## 1. Introduction

During pregnancy, the immune system plays a crucial role in regulating the interactions between the fetus and the mother to ensure maternal health and healthy fetal development. The local immune environment of the reproductive tract facilitates fetal implantation and growth. In concert with local immune modifications, systemic immune responses also undergo adaptations to protect both the mother and fetus from infections in addition to preventing detrimental maternal immune responses towards the semi-allogeneic fetus. As key modulators of immune response, cytokines in the local tissue microenvironment and in the peripheral blood have been extensively examined in regard to their levels and biological functions [1,2,3]. Accumulating evidence suggests that maternal blood cytokine profiles can reveal immune modifications that promote successful pregnancy as well as dysregulated immune responses that cause pregnancy complications or abnormal fetal development [2,4].

Serum cytokine patterns have been reported to reflect the dynamic changes in the maternal immune status over the course of a healthy pregnancy. A study of nineteen cytokines in 1149 longitudinal serum samples from 707 pregnant women showed that inflammatory cytokines peaked in the first trimester, decreased in the second trimester, and increased again at the end of the third trimester, such as IL-1β, IL-6, and IL-8 [5]. The anti-inflammatory cytokines showed more diverse patterns. For example, IL-9 decreased throughout pregnancy while IL-13 peaked near full-term. In the same dataset, several chemokines showed a clear trend of decrease from first trimester to term, particularly eotaxin [5]. More importantly, changes in cytokine patterns were associated with maternal obesity and smoking [5]. Many other smaller studies also described the potential of maternal serum cytokine profiles as biomarkers to monitor healthy pregnancy or to detect the early onset of pregnancy-related complications before clinical recognition [6,7].

Preeclampsia, a pregnancy complication involving chronic abnormal immune activation, has been associated with increased levels of inflammatory cytokines in maternal serum [8,9,10]. It has been reported that TNF-*α*, IFN-*γ*, IL-2, IL-8, and IL-6 are present at significantly higher levels in women with preeclampsia compared to women with unaffected pregnancies [8]. Lower levels of IL-10 in early pregnancy may be associated with the development of preeclampsia later on in gestation [8]. These studies have provided opportunities to further develop biomarkers for diagnosing pregnancy-related pathologies like preeclampsia. However, maternal health conditions, variation in baseline levels, and gestational age can also significantly affect concentrations of these cytokines in maternal blood [5]. Thus, use of single cytokines as accurate biomarkers is limited by lack of specificity.

In the present pilot study, we characterized the longitudinal serum cytokine patterns during healthy pregnancy using a much larger array of 96 cytokines. Since a single cytokine may have limited value in characterizing the immune status of the mother, as a proof-of-concept testing, we developed integrated cytokine panels that have potential to improve the characterization of immune signatures at different stages of healthy pregnancy. We also evaluated the potential of using integrated cytokine scores for identifying pathological conditions (e.g., preeclampsia) before clinical signs are present. The combined effects of serum cytokines on trophoblast migration were assessed using an in vitro cell migration model. The goal of this work is to explore new approaches of developing non-invasive biomarker panels for immune profiling and early detection of pathological conditions during pregnancy. The integrated cytokine profiles also provide additional insight into the immunological regulation that supports healthy pregnancy.

## 2. Materials and Methods

### 2.1. Study Cohort

Serum samples were prospectively and longitudinally collected from pregnant women at four timepoints during pregnancy (10–12 weeks, 18–20 weeks, 26–28 weeks and 34–36 weeks) as part of the Yale University Reproductive Sciences Pregnancy Study (YURS-Pregnancy Study, IRB Approval number 1309012696). Subjects with uncomplicated pregnancies were retrospectively reviewed and selected for the current study as the “healthy pregnancy” cohort (n = 25). Subjects with preexisting health issues (e.g., preexisting diabetes mellitus, chronic hypertension, autoimmune disorders, coagulation or bleeding disorders), those on medications other than prenatal vitamins and those with pregnancy complications (including but not limited to hypertensive disorders of pregnancy, gestational diabetes, preterm delivery, placenta accreta spectrum, large or small for gestational age newborns, multiples and pregnancies affected by fetal anomalies), as well as pregnancies conceived via in vitro fertilization (IVF), were excluded from the study. Additionally, four subjects who developed preterm preeclampsia and delivered at 31–36 weeks of gestation for preeclampsia-related indications were identified through chart review. Preeclampsia was defined by the American College of Obstetricians and Gynecologists (ACOG) criteria including at least two episodes of new-onset hypertension (systolic blood pressure > 140 mmHg or diastolic blood pressure > 90 mmHg) with new-onset proteinuria after 20 weeks of pregnancy. Serum specimens from these subjects were collected at 20 weeks of gestation before clinical evidence of preeclampsia emerged. These subjects represented the “subsequent preeclampsia” group. A demographic table is included in Appendix A.

### 2.2. Serum Sample Preparation

Venous blood was collected in tubes without anticoagulants and was allowed to clot for 30 min at room temperature, after which the samples were centrifuged at 1000 *g*. The serum was then transferred into a clean cryotube, flash-frozen in liquid nitrogen, and stored at −80 degrees Celsius until further processing. Freeze–thaw cycles were avoided.

### 2.3. Serum Cytokine Multiplex Assay

Serum samples were analyzed by Eve Technologies (Calgary, AB, Canada) using a Human Cytokine/Chemokine 96-Plex Discovery Assay Array (catalog number HD96) with a Luminex^®^ 200 platform.

### 2.4. Cell Culture and Transwell Migration Assay

The trophoblast cell line Sw.71 was propagated in RPMI 1640 medium containing 10% fetal bovine serum (FBS), L-glutamine, and penicillin/streptomycin. All cells were cultured in an incubator at 37 °C (5% CO_2_). A transwell migration assay was conducted per the manufacturer’s instruction using the 8 μm fluorometric format CytoSelect 96-well cell migration assay kit (Cell Biolabs, San Diego, CA, USA). Briefly, 100 μL suspension of 0.5 × 10^6^ cells/mL in serum-free RPMI medium was placed in the upper chamber, while 150 μL of serum-free medium containing 5% of the corresponding human serum was added to the lower chamber. The cells were allowed to migrate for 24 h. After this period, a cell detachment reagent was applied, and the number of migrated cells was quantified using a cell titer luminescence assay. The relative luminescence was determined using a Navigator Microplate luminometer plate reader (Promega, Madison, WI, USA).

### 2.5. Statistical Analysis

Samples were processed in two batches. A subset of samples were included in both batches to facilitate batch correction. Cytokine concentrations were calculated using standard curves in the assay. Data were then batch-corrected and normalized for further analysis. Batch correction and normalization was performed in one step by normalizing the data for each timepoint for each gene to the average of that gene’s values across all samples included in that batch at timepoint 0 (~12 wks): $C_{g,t,norm}=C_{g,t}/\frac{\sum_{Batch}C_{g,t0}}{N_{Batch}}$ [*C* = concentration; *g* = gene; *t* = timepoint; *t*0 = timepoint 0 (~12 wks); *N_Batch_* = number of samples in batch]. Cytokine concentration data of the 21 patients for whom all four timepoints were analyzed were split into three separate groups based on their individual longitudinal trends after outlier removal. *p*-values were calculated using one-way ANOVA and displayed as −log10 values. Cytokine data from thirteen healthy women were randomly selected from our dataset. Using this subset of 96-cytokine data, we identified a panel of 21 cytokines for T-distributed stochastic neighbor embedding (tSNE) allowing for visually distinguishing samples collected at different timepoints. These 21 cytokines were selected for inclusion as the top ten up-trending cytokines and the top eleven down-trending cytokines throughout the gestational period. Cytokine data from a separate subset of thirteen healthy women were then used to perform the same tSNE analysis using the previously identified 21-cytokine panel. Data of five cytokines (I-TAC, MCP-4, MPIF-1, IL-8 and perforin) were integrated using a weighted linear combination of batch-corrected and z-score standardized concentration values. Cytokine contribution weights were determined using multiple linear regression analysis based on available data for four preeclampsia samples. Statistical analysis was performed using GraphPad Prism 9 and custom python scripts.

## 3. Results

### 3.1. Serum Cytokines with Longitudinal Trends During Healthy Pregnancy Were Identified in the Cytokine 96-Plex Screening

**Figure 1:**
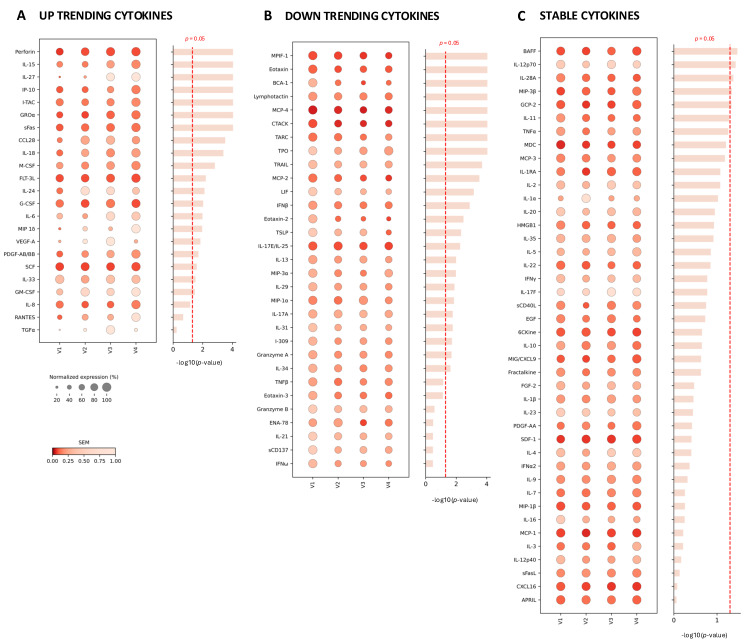
Overview of the longitudinal trends in 96-plex cytokine profiling of serum samples collected at four timepoints of healthy pregnancy. (**A**) Up-trending cytokines. (**B**) Down-trending cytokines. (**C**) Cytokines with stable patterns. Data shown as normalized concentrations in dot plots where the size corresponds to the normalized concentration and the color represents the standard error of the mean (SEM) for each timepoint across 21 healthy pregnant women. *p* < 0.05, as assessed using ANOVA with timepoints as variables.

The trend of each cytokine level over the course of a healthy pregnancy at four timepoints was analyzed based on the data of 21 subjects (Figure 1). The individual cytokine concentration value was normalized to the corresponding concentration of the first timepoint. First, the levels of 23 cytokines increased over the course of the pregnancy. Among these, the timepoints of blood collection significantly impacted the serum levels of twenty cytokines (Figure 1A, *p* < 0.05). Among these up-trending cytokines, VEGF-A and TGFα levels were increased at the first three timepoints and significantly decreased at the last timepoint; IL-24 and CCL28 levels also slightly decreased at the last time point. On the other hand, the levels of 31 cytokines decreased over the course of the pregnancy, of which 24 cytokines were significantly impacted by the collection timepoints (Figure 1B, *p* < 0.05). Among the down-trending cytokines, TPO and MIP-3α levels slightly increased at the last timepoint. Finally, we found that the levels of 42 cytokines remained generally stable throughout the pregnancy with some minor fluctuations between various timepoints (Figure 1C).

**Figure 2:**
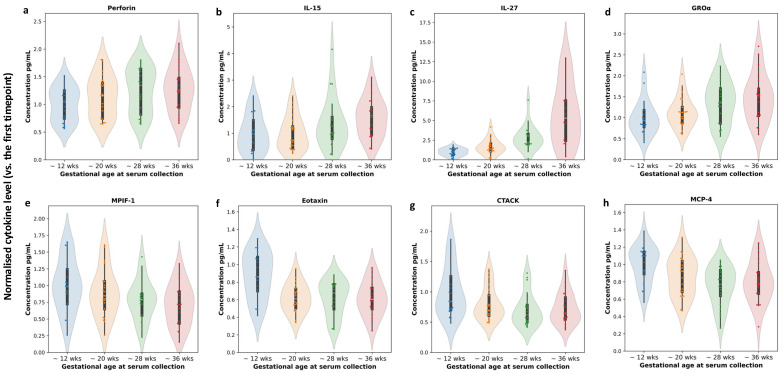
Representative up- and down-trends in serum cytokine levels of healthy pregnant women. (**a**) Perforin; (**b**) IL-15; (**c**) IL-27; (**d**) GROα; (**e**) MPIF-1; (**f**) Eotaxin; (**g**) CTACK; (**h**) MCP-4. All represented trends are significant using repeated measures ANOVA test at *p* < 0.0001. X-axis: timepoints of serum collection during pregnancy; Y-axis: relative serum cytokine levels normalized to the first timepoint.

Perforin was identified as an up-trending cytokine (Figure 2a). Our data also shows the upregulation of IL-15 (Figure 2b). These trends are consistent with previous observations [5]. Additionally, our data demonstrates the longitudinal gradual increase in serum IL-27 during healthy pregnancy (Figure 2c). The serum level of IL-27 was reported as higher in healthy women at 30–34 weeks gestation in comparison with non-pregnant women [11]. GROα, also known as CXCL1, a main ligand to CXC motif chemokine receptor 2 (CXCR2), was also one of the top up-trending cytokines (Figure 2d). GROα is extensively produced by the cervix and minimally in trophoblast tissue. Its receptor CXCR2 is present in relatively low levels in the placenta. Lower GROα levels in maternal serum have been associated with spontaneous preterm birth and implicated in fetal membrane inflammation in the setting of bacterial stimuli [12].

Down-trending cytokines include MPIF-1 (CCL23, Figure 2e), eotaxin (Figure 2f), CTACK (CCL27, Figure 2g), and MCP-4 (Figure 2h). The down-trend of serum eotaxin throughout gestation is consistent with previous observations [5].

### 3.2. A Panel of Twenty-One Cytokines Can Characterize the Longitudinal Changes in Serum During Pregnancy

**Figure 3:**
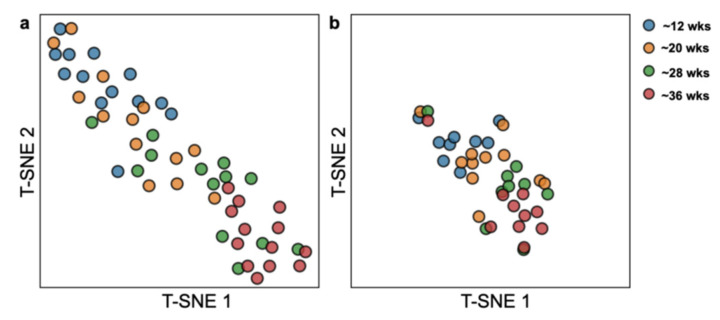
T-distributed stochastic neighbor embedding (tSNE) of two serum sample sets based on a 21-cytokine panel. (**a**) tSNE plot of 21 cytokines can distinguish samples collected in early pregnancy (<24 weeks) from those collected in later pregnancy (>24 weeks). Data of 96 cytokines in 52 serum samples from 13 randomly selected pregnant women at four timepoints were used to optimize and generate the 21-cytokine panel. (**b**) tSNE plot of the 21-cytokine panel using a different set of data from another 11 pregnant women.

**Figure A1:**
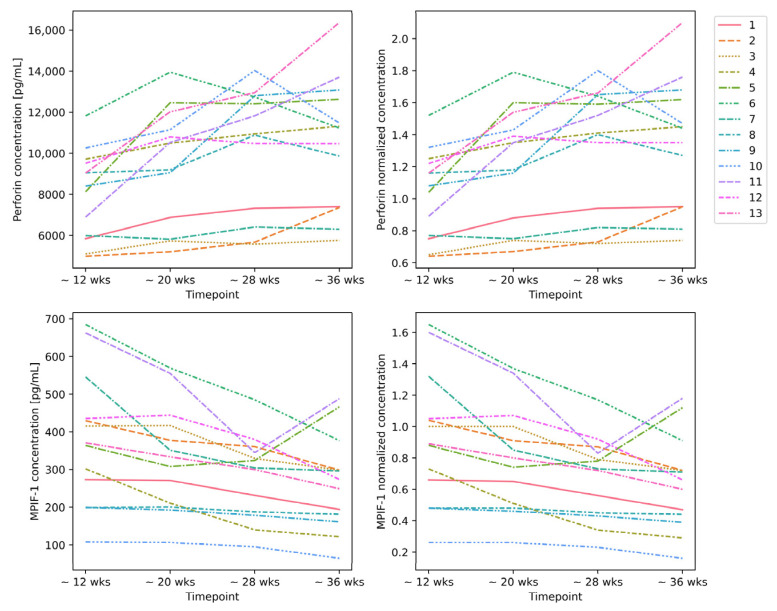
Longitudinal trends of Perforin and MPIF-1 in absolute (pg/mL) and normalized concentration values.

The trend of any single cytokine provides limited information about the complex changes in serum during pregnancy. Therefore, we determined the extent to which a panel of cytokines could improve the profiling of longitudinal serum samples. Principal component analysis was performed using the cytokine data of 52 serum samples collected from thirteen randomly selected pregnancy women at four timepoints. From the originally analyzed 96 cytokines, we narrowed down to a panel of twenty-one cytokines, which allowed us to use T-distributed stochastic neighbor embedding (tSNE) to visually distinguish samples collected at different timepoints of the pregnancy (Figure 3a). The twenty-one cytokines in this panel were selected to include the ten most significantly up-trending cytokines as shown in Figure 1a (perforin, IL-15, IL-27, IP-10, I-TAC, GROα, sFas, CCL28, IL-18, and M-SCF) and the eleven most significantly down-trending cytokines as shown in Figure 1b (MPIF-1, eotaxin, BCA-1, lymphotactin, MCP-4, CTACK, TARC, TPO, TRAIL, MCP-2, and LIF). Example plots demonstrating raw concentration values and associated normalized values for the most significantly up-trending and down-trending cytokines are noted in Appendix A Figure A1. To assess the reliability of this 21-cytokine panel, we performed the same tSNE analysis on a different set of samples from another eleven pregnant women (Figure 3b). Again, we were able to distinguish samples collected in the early stage (<24 weeks) from those collected in the late stage of the pregnancy (>24 weeks).

### 3.3. Maternal Serum Promotes Trophoblast Migration in an In Vitro Model, in Which the Migration-Promoting Activity of Serum Increases with Advancing Gestational Age

**Figure 4:**
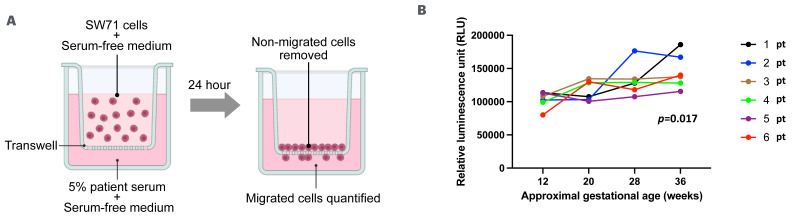
Migration of trophoblast cells induced by serum samples collected at four timepoints of healthy pregnancy.  (**A**) Schematic representation of the transwell migration system incorporating trophoblast cell line SW71 and maternal serum samples. (**B**) Quantification of SW71 cells that migrated in 24 h through the transwells. Each colored curve represents a set of serum samples collected from one individual woman at different gestational ages. *p* < 0.05, as assessed using ANOVA with timepoints as the variable.

In our longitudinal serum cytokine analysis, several potent chemotactic molecules showed trends of increase or decrease. Some of them, such as the up-trending IP-10, IL-15 and IL-6, or the down-trending MPIF-1 and eotaxin, have been individually implicated in promoting the migration of trophoblast cells [13]. Using an in vitro cell migration model (Figure 4A), we evaluated the combined effect of maternal serum factors on trophoblast cell migration in a longitudinal manner. Maternal serum collected at advanced gestational ages resulted in a statistically significant increase in trophoblast migration compared to serum collected in the first trimester (Figure 4B, *p* < 0.05). The combined effect of serum factors in the longitudinal maternal serum samples indicates an overall increasing trend in promoting trophoblast migration.

### 3.4. I-TAC, MPIF-1 and IL-8 Are Potential Serum Markers for Predicting Preterm Preeclampsia

**Figure 5:**
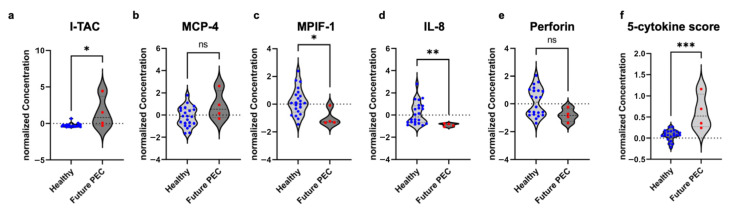
Maternal serum cytokine levels at 20 weeks of gestation. Serum samples were collected from healthy women (n = 23) and women who subsequently developed preterm preeclampsia in the third trimester (n = 4). Notably, none of the subjects exhibited signs of preeclampsia at the time of serum collection. Graphs include normalized levels of five individual cytokines (**a**–**e**) and integrated scores of the five cytokines (**f**). * *p* < 0.05, ** *p* < 0.005, *** *p* < 0.0005, or not significant (ns) as determined by Wald test.

Typically, women who will develop preterm preeclampsia do not present any clinical signs prior to 20 weeks gestation and often much later. However, using the serum samples collected at 18–20 weeks of pregnancy, we identified several cytokines with different concentrations in the serum of healthy pregnancies compared to those who subsequently developed preeclampsia. Specifically, the levels of I-TAC (Figure 5a, *p* = 0.016) and MCP-4 (Figure 5b, *p* = 0.15) were higher while the levels of MPIF-1 (Figure 5c, *p* = 0.016), IL-8 (Figure 5d, *p* = 0.0049), and perforin (Figure 5e, *p* = 0.082) were lower in the serum of patients who ultimately developed preeclampsia in comparison with healthy controls at matched gestational ages. Notably, at the time of serum collection all subjects were managed as healthy pregnancies since none had yet exhibited clinical signs of preeclampsia. By combining the results of these five factors to generate an integrated five-cytokine score, we were able to use serum samples from 18 to 20 weeks of gestation to differentiate asymptomatic pregnant subjects who later went on to develop preterm preeclampsia from those who continued to have healthy pregnancies (Figure 5f, *p* = 0.00049).

## 4. Discussion

This study characterized longitudinal changes of 96 cytokines in maternal blood during normal pregnancy and revealed temporal patterns of immunologic changes, supporting the hypothesis that the maternal serum cytokine profile reflects the “immune clock” of human pregnancy. We demonstrated that maternal serum has increasing migration-inducing effect on trophoblast cells over the course of pregnancy. Finally, a scoring system based on the levels of five selected cytokines was developed to predict preeclampsia well before clinical onset. The strengths of this study include the simultaneous assessment of 96 cytokines, the coverage throughout early to late stages of pregnancy using blood samples collected at four timepoints (10–12 weeks, 18–20 weeks, 26–28 weeks, and 34–36 weeks) within the same patients, and the application of statistical techniques for generating integrated cytokine panels and multi-cytokine scores.

While prior research has described the relevance of individual cytokines in this 21-cytokine panel to pregnancy, we are yet to identify a clear connection between them. It is possible that each of them reflects different aspects of the immunological changes in pregnancy. Perforin, identified as one of the up-trending cytokines (Figure 2a) is known to be expressed by uterine natural killer (NK) cells [14,15]. The abundance of perforin has also been detected at the maternal–fetal interface [16]. Another one of our identified up-trending cytokines, IL-15, is abundant in the uterus during pregnancy and plays a critical role in regulating uterine natural killer cell differentiation [17]. IL-15 deficiency in a mouse model led to growth restriction, abnormal decidua, and impaired remodeling of spiral arteries [18,19]. Both mRNA and protein levels of IL-15 were significantly reduced in preeclamptic placental tissues compared to normal controls [20]. However, several studies showed elevated levels of IL-15 in serum of women affected by preeclampsia [10,21,22]. The upregulation of IL-15 mRNA and protein was also detected in placentas from miscarriages, especially in patients with recurrent early pregnancy loss [23]. Another of the up-trending cytokines, IL-27, is primarily produced by antigen-presenting cells in response to activation of Toll-like receptor and interferon-gamma. IL-27 has both pro- and anti-inflammatory actions that are critical for trophoblast proliferation, invasion and migration [24,25]. Previous studies suggest that during pregnancy, extravillous trophoblasts, syncytiotrophoblasts, and decidual cells produce increasing levels of IL-27, resulting in increasing levels in the placenta and in maternal serum [25,26]. Reduced IL-27 expression in the decidual cells was associated with early pregnancy loss, while increased IL-27 levels in maternal serum have been implied in miscarriages, preterm birth, and preeclampsia [27].

**Figure A2:**
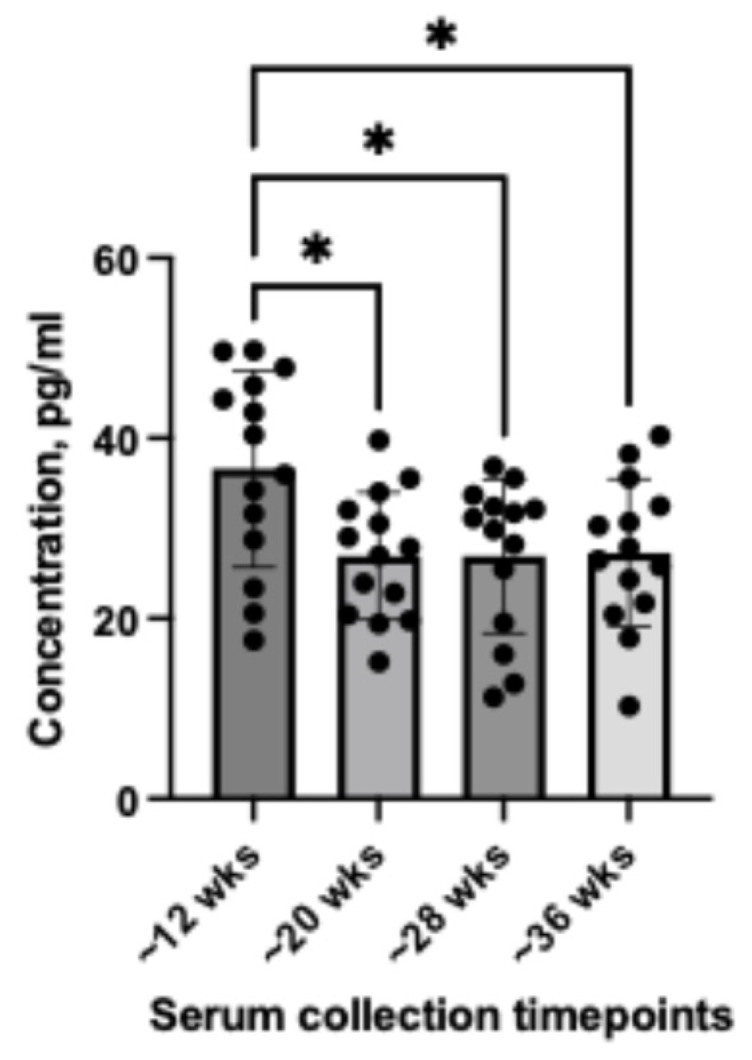
Absolute concentration of eotaxin over the course of pregnancy. * *p* < 0.05.

A clear down-trend in serum eotaxin throughout gestation was previously reported in a study of 1149 longitudinal serum samples from 707 pregnant women [5]. This study also suggested that clinical parameters have minimal impact on the decreasing trend of eotaxin in maternal serum; thus, decreasing eotaxin may be a robust hallmark of normal pregnancy. Our data validated this longitudinal trend of serum eotaxin during normal pregnancy. It has been reported in a machine learning-based analysis of multiple serum cytokines that the level of serum eotaxin was significantly altered in a group of pregnancies affected hypertensive disorders, which again supports the potential of eotaxin as a biomarker for monitoring pregnancy [28]. In our study eotaxin was one of the cytokines that did not demonstrate a batch effect thus absolute concentrations were also reviewed. Our values were strikingly comparable with the values from Varghese et al. manuscript [28]. In that manuscript, mean eotaxin serum concentration in healthy women at 38 weeks was 28.2 pg/mL (IQR 18.89–38.98), while in our study mean concentration at 36 weeks was 27.29 pg/mL (SD ± 8.19) (Appendix A Figure A2). The authors of Jarmund et al. manuscript report absolute concentration as mean over all pregnancy timepoints 88.7 pg/mL (IQR 68.3–113.7). While direct comparison is not possible given this value representing all gestational ages combined, this concentration appears higher than our values at any point in pregnancy. We suspect that this may be related to the variability between the labs. Thus, if this is the case, each lab will need to develop their own reference system, similarly to how it is currently being done for maternal serum alpha-fetoprotein (lab-specific Multiples of the Median (MoM)). Alternative would be for a patient to have two blood draws overtime (e.g., at 12 weeks and then 20) for individualized normalization.

CTACK (cutaneous T-cell-attracting chemokine, also known as CCL27) was another down-treading cytokine identified in our analysis. A prior study compared the levels of CTACK in plasma samples of women experiencing premature pre-labor rupture of membranes (PPROM) with normal control plasma samples [29]. Women with PPROM had significantly lower levels of plasma CTACK at the time of admission for PPROM than the gestational age-matched healthy controls. This observation in conjunction with our results suggests that premature decline of CTACK levels in maternal blood may reflect an immunological dysregulation associated with abnormal labor initiation and progression, particularly preterm birth with PPROM.

In this study, we utilized an in vitro trophoblast cell migration model to evaluate the effect of maternal serum on trophoblast migratory behavior. As we expected, maternal serum induced the migration of trophoblast cells. We originally anticipated that the ability of maternal serum to stimulate trophoblast migration would decrease towards the end of pregnancy, since it is believed that trophoblast migration mostly stops in the second trimester. Unexpectedly, the results of this model suggest that the migration-inducing activity of maternal serum factors consistently increases throughout pregnancy, including in the third trimester and at term. This observation leads to several hypotheses. One hypothesis is that as healthy pregnancy progresses, the migration-inducing activity of maternal blood increases while the decidua is also increasingly able to restrain the migration of trophoblasts. Alternatively, it is also possible that trophoblast cells gradually lose their ability to migrate and invade as the pregnancy advances. These changes maintain a tightly controlled balance to ensure healthy development of the placenta. Another explanation is that the maternal serum contents do not represent the local cytokine signals at the maternal–fetal interface. Indeed, this model has its limitations since it is only a simplified in vitro system that does not incorporate all the local interactions at the maternal–fetal interface. Despite these limitations, this model serves as a tool for functional profiling of maternal serum factors. Modifications of this model (e.g., addition of a neutralizing antibody against a specific cytokine or modulators of specific molecular pathways) allow for studies specific to the functions of individual factors and molecular pathways in regulating trophoblast migration.

There have been many attempts to develop biomarkers to identify women at risk of preeclampsia, including the utility of sFLT/PlGF ratio in maternal serum [30,31,32]. A cut off for this ratio of 38 has achieved excellent negative predictive values of 98% and 94% for preeclampsia development within 2 and 4 weeks, respectively. However, depending on the tested population and predicted time of preeclampsia onset, the positive predictive value varies from 6 to 77% [33,34]. Other cut-offs and combinations with other clinical or laboratory parameters have been evaluated [35,36,37], but clinical implementation in the US population is still limited by high cost, lack of validation, and other obstacles [38]. Cytokine panels like the one identified in our study should be further examined as a stand-alone panel or as part of a multimodal screening tool in a larger population. Our multi-cytokine scoring method provides another new direction for developing prediction tests for preeclampsia and other complications of pregnancy many weeks prior to clinical signs and diagnosis.

One main limitation of this study is a relatively small sample size due to the cost and sample availability. In the longitudinal analysis, serum samples were collected from 21 subjects, which limited the feasibility to evaluate potential impacts of physiological or lifestyle factors on the cytokine pattern. For example, in the study of 707 pregnant women [5], differences in smoking, body mass index, fetal sex, and birth weight were associated with distinct patterns of maternal cytokine profiles. In another study of 222 women, maternal serum levels of B-cell activating factor (BAFF) gradually decreased between the first and third trimesters followed by subsequent increase in healthy pregnancies [39]. Subjects who subsequently developed preeclampsia showed significant increase in the level of serum BAFF. However, our data for BAFF did not show any obvious trend (Figure 1C). Expansion of our study to a larger number of subjects and including more serum samples from pregnancies with complications will allow us to further define the integrated cytokine panel and to better evaluate the translational potential of the panel.

## 5. Conclusions

Common pregnancy complications including hypertensive disorders of pregnancy, fetal growth restriction, and placenta accreta spectrum disorders have been associated with inflammation, abnormal trophoblast invasion, and suboptimal remodeling of spiral arteries. Blood marker-based non-invasive early detection or prediction tests are urgently needed to assist with clinical decision-making and improve the clinical outcomes of pregnancies affected by these complications. A better understanding of the normal maternal cytokine profile at different stages of pregnancy is a critical step towards developing these tests. Our study demonstrates that the “immune clock” of human pregnancy is trackable through integrating a panel of 21 serum cytokines or chemokines (out of the 96 candidate molecules) using data dimensionality reduction techniques. The identification of this serum cytokine panel set a foundation for future studies in a larger cohort of serum samples collected during healthy and pathological pregnancies, which will further improve the potential for clinical translation of this approach.

## Data Availability

Data and materials described in this manuscript will be made available upon request for non-commercial use, without compromising participant confidentiality.

## Abbreviations

The following abbreviations are used in this manuscript:

TNF-*α*	Tumor necrosis factor alpha
IFN-*γ*	Interferon-gamma
IL	Interleukin
VEGF-A	Vascular endothelial growth factor A
TGFα	Transforming growth factor alpha
TPO	Thrombopoietin
MIP-3α	Macrophage inflammatory protein-3 alpha
MPIF-1	Myeloid progenitor inhibitory factor 1
MCP-4	Monocyte chemotactic protein 4, CCL13
CTACK	Cutaneous T-cell attracting chemokine, CCL27
IP-10	Interferon-gamma-induced Protein 10 kDa, CXCL10
BAFF	B-cell activating factor
I-TAC	Interferon-inducible T-cell Alpha Chemoattractant, CXCL11
sFLT	Soluble fms-like tyrosine kinase-1
PlGF	Placental growth factor
GROα	Growth-regulated oncogene-alpha, CXCL1
sFas	Serum soluble Fas (sFas), a circulating isoform of Fas receptor (CD95)
CCL28	C-C motif chemokine ligand 28
M-SCF	Macrophage colony-stimulating factor

## Appendix A

**Table A1:** Demographic table of the study cohort.

	Healthy Pregnancy (N = 25)	PEC Pregnancy (N = 4)
**Age at delivery (SD)**	31 (3.91)	31 (5.4)
**Demographic**		
**Race**		
White	21	1
Black or African American	1	3
Asian	3	0
**Ethnicity**		
Hispanic or Latino	3	0
Non-Hispanic	22	4
**BMI**		
Pre-pregnancy (SD)	26 (6.70)	33 (6.21)
At delivery (SD)	31 (7.17)	37 (6.75)
**Gestational age at the delivery, weeks (SD)**	40.00 (1.25)	35.79 (2.44)
**Mode of delivery, C-section**	11	3
**Newborn sex**		
Male	10	4
Female	15	0
